# Exploring the Feasibility of Integrating Cardiopulmonary Exercise Testing, Echocardiography, and Biomarkers for Predicting Atrial Fibrillation Recurrence: Rationale, Design and Protocol for a Prospective Cohort Study (The PLACEBO Trial)

**DOI:** 10.3390/jcm14051690

**Published:** 2025-03-03

**Authors:** Aristi Boulmpou, Theodoros Moysiadis, Georgios Zormpas, Eleftherios Teperikidis, Vassilios Vassilikos, Georgios Giannakoulas, Christodoulos Papadopoulos

**Affiliations:** 1 Third Department of Cardiology, Ippokratio General Hospital, Aristotle University of Thessaloniki, 54642 Thessaloniki, Greece; 2 Department of Computer Science, School of Sciences and Engineering, University of Nicosia, Nicosia 2417, Cyprus; 3 Second Department of Cardiology, Ippokratio General Hospital, Aristotle University of Thessaloniki, 54642 Thessaloniki, Greece; 4 First Department of Cardiology, AHEPA University Hospital, Aristotle University of Thessaloniki, 54636 Thessaloniki, Greece

**Keywords:** atrial fibrillation, cardiopulmonary exercise testing, echocardiography, personalized care

## Abstract

**Background**: Paroxysmal atrial fibrillation (PAF) presents significant challenges in management due to its unpredictable recurrence and associated complications. Current prognostic tools lack precision in predicting PAF outcomes, highlighting the need for a comprehensive approach integrating multiple diagnostic modalities. **Methods:** The PLACEBO pilot study is a prospective observational investigation enrolling 80 adults with PAF from a tertiary hospital. Baseline assessments include cardiopulmonary exercise testing (CPET), a complete echocardiographic evaluation, 24-h ambulatory electrocardiographic monitoring, and plasma biomarker analysis. Participants will be followed for 12 months, with the primary outcome being AF recurrence. Secondary outcomes include cardiovascular events and other clinical indicators relevant to disease progression. **Results:** The study will assess the feasibility of integrating CPET parameters, echocardiographic indices, and plasma biomarkers into clinical practice for prognostic evaluation. Data analysis will focus on identifying potential associations between these modalities and PAF recurrence, contributing to improved risk stratification. **Conclusions**: By integrating CPET, echocardiographic parameters, and plasma biomarkers, the PLACEBO pilot study aims to enhance risk stratification and improve the prognostic assessment of patients with PAF. The findings from this study may pave the way for future research, ultimately contributing to the development of personalized management strategies.

## 1. Introduction

Atrial fibrillation (AF) is the most common cardiac arrhythmia encountered in the Western world, constituting a significant burden on patients and healthcare systems [1]. AF is characterized by an irregular and often rapid cardiac rhythm and is associated with significant morbidity and mortality, including increased risks of stroke, heart failure, and all-cause mortality [2]. Despite advancements in the management and of AF and in therapeutic approaches, the prevalence of AF continues to rise and is accompanied by an increase in the incidence of major adverse cardiovascular events (MACE), underscoring the need for novel, patient-centered management strategies [3].

Paroxysmal atrial fibrillation (PAF), a subtype of AF, is defined by self-terminating episodes that usually last less than 7 days [4]. Despite its transient nature, PAF poses substantial challenges in clinical management due to its multifactorial basis, unpredictable recurrence, and associated complications [5,6]. The prognosis of PAF varies depending on several factors, including the frequency and duration of episodes, the presence of underlying cardiovascular conditions, and individual patient characteristics [7]. While PAF generally carries a lower risk of adverse outcomes such as stroke compared to persistent or permanent AF, it still necessitates high awareness and effective management to mitigate potential risks and optimize long-term outcomes [8]. Understanding the natural history and prognostic indicators specific to PAF is crucial for tailoring therapeutic approaches aimed at rhythm control, reducing symptoms, and minimizing the risk of MACE in affected individuals.

Cardiopulmonary exercise testing (CPET) is a valuable tool in our clinical practice, providing comprehensive information about the cardiovascular, respiratory, and musculoskeletal response to exercise [9,10]. CPET plays a crucial role in predicting future cardiac events in various conditions, including AF [11]. However, data regarding the precise relationship between cardiorespiratory fitness and AF prognosis are conflicting, with some studies suggesting that higher fitness levels increase AF risk and others indicating a protective effect [12,13]. Recent advancements in CPET have opened new avenues for the prognostic evaluation of AF [14].

In the same line, novel echocardiographic indices and plasma biomarkers have emerged as promising tools for assessing cardiac function and inflammation, critical components of the pathophysiology of AF [15,16,17,18]. These advancements highlight the need for an integrative approach to AF prognostication, incorporating CPET, echocardiography, and biomarkers to address current knowledge gaps.

We set out to design a pilot study with the aim of integrating the aforementioned innovative diagnostic approaches in AF management to explore their feasibility and potential utility for the prediction of arrhythmic recurrences. Therefore, the PLACEBO trial serves as an exploratory feasibility study to evaluate the potential significance of these diagnostic modalities in predicting AF recurrence and related adverse outcomes. By providing initial insights, the trial seeks to identify promising predictors and generate hypotheses that could guide future, larger-scale studies aimed at enhancing predictive accuracy and supporting more personalized management strategies for patients with PAF.

## 2. Methods

### 2.1. Study Design

The PLACEBO (“ParoxysmaL Atrial fibrillation prognosis based on Cardiopulmonary Exercise test data and novel echocardiographic and plasma BiOchemical indices”) trial is a pilot, prospective, observational cohort study designed to explore the feasibility of integrating CPET data, novel echocardiographic indices, and plasma biochemical markers in consecutive patients with PAF for the prediction of AF recurrences. As this is a feasibility study, no specific treatment interventions were prescribed as part of the study protocol. Instead, the study aims to identify potential signals and generate hypotheses for future larger-scale investigations.

The sample size of 80 participants was chosen pragmatically, based on the feasibility of recruitment and data collection within the study’s timeframe and resources. Formal sample-size calculations were not performed, as the primary aim of the study is to assess the feasibility of the proposed methodology and to explore potential predictors of AF recurrence, rather than to test specific hypotheses. This approach aligns with the exploratory nature of pilot studies, aiming to provide preliminary insights into the utility of these diagnostic modalities and establish a basis for future, larger-scale studies.

The study is being conducted at Ippokratio General Hospital in Thessaloniki, Greece. The protocol of the trial is registered at ClinicalTrials.gov (Identifier: NCT05246423) and has been reviewed and approved by the Institutional Review Board (IRB) of Ippokratio General Hospital, as well as by the Aristotle University of Thessaloniki Ethics Committee. Written informed consent was obtained from all participants prior to any study-related procedures. The study is being conducted in accordance with the Declaration of Helsinki and Good Clinical Practice guidelines.

### 2.2. Participants

Participants in the study were adults aged 18 years and older, diagnosed with PAF, and in sinus rhythm at the time of baseline evaluation. Eligible participants were required to undergo CPET and comply with follow-up requirements.

PAF was defined according to the ESC 2020 and 2024 Guidelines as AF episodes that terminate spontaneously or with an intervention within 7 days, typically within 48 h [4,19]. Patients with persistent or permanent AF were excluded to maintain a homogeneous study population. To ensure accurate classification, all patients had a documented time of AF onset, as reported from one of the following sources:

- Hospitalization with a recorded AF episode and documented termination.
- Outpatient or patient visit to the Emergency Department with a definite AF onset time, either self-reported or confirmed via surface electrocardiogram (ECG) or 24-h ECG Holter monitoring.
- AF episode terminated in-hospital, either spontaneously, pharmacologically, or electrically.

Additionally, all participants underwent 24-h ambulatory ECG Holter monitoring at baseline to confirm sinus rhythm and assess AF burden. Medical records and prior ECG documentation were reviewed to validate AF classification and exclude cases of persistent AF.

To assess the duration of AF, data were collected regarding the time since the first documented AF diagnosis based on medical records and patient history. While the exact onset date was not necessarily available, disease duration was recorded as a key variable to assess AF burden and progression.

The inclusion criteria were meeting these conditions and being capable of providing written informed consent. The exclusion criteria consisted of structural cardiomyopathy, congenital heart disease, permanent AF, recent acute coronary syndrome (within the last month), heart failure (HF) with reduced ejection fraction, end-stage renal disease, autoimmune diseases, active malignancies, uncontrolled thyroid disease, recent surgery (within the last 2 months), poor echocardiographic windows, uncontrolled systemic hypertension, prior catheter ablation for AF, and pregnancy. Patients unable to comply with the study schedule or provide informed consent were also excluded.

### 2.3. Data Collection

#### 2.3.1. Baseline

Participants underwent a comprehensive assessment at baseline, which included the collection of demographic data and full medical history, CPET, full transthoracic echocardiographic assessment, 24-h ECG ambulatory Holter monitoring, and measurement of plasma biomarkers.

1. Demographics and baseline characteristics: The baseline characteristics of the study population were collected and will be summarized using descriptive statistics. Demographic data (age, gender, body mass index), clinical characteristics (history of cardiovascular diseases, medication use), and baseline values of CPET parameters, echocardiographic indices, and plasma biomarkers will be presented. Continuous variables will be expressed as means and standard deviations or medians and interquartile ranges, depending on their distribution. Categorical variables will be presented as frequencies and percentages.
2. CPET: The study participants underwent a full CPET workup, including a symptom-limited exercise test on a cycle ergometer, with continuous 12-lead ECG monitoring of cardiac rhythm, measurement of respiratory gases, and assessment of heart rate (HR) and arterial blood pressure. CPET allows for the measurement of over 50 parameters, each of which holds prognostic significance across a wide range of cardiovascular conditions. The primary measures obtained from CPET include peak oxygen uptake (peak VO_2_) and the minute ventilation/carbon dioxide production slope (VE/VCO_2_ slope), indices whose prognostic role has already been established in HF. Gas-exchange physics in patients with AF have been an item of interest for decades; nevertheless, the exact role of CPET in determining the AF prognosis has not yet been established.
3. Transthoracic echocardiography: A standard, complete transthoracic echocardiographic study was performed in all patients under stable hemodynamic conditions. All echocardiograms were conducted by a single experienced cardiologist using a GE Vivid E95 ultrasound system (GE Healthcare, Chicago, IL, USA). A series of echocardiographic parameters were obtained, including the dimensions and functionality of the left ventricle (LV) [volume, mass, dimensions, left ventricular ejection fraction (LVEF) as measured using Simpson’s method, and global longitudinal strain (GLS)], diastolic indices of the LV (E/A, E/E’ ratio, mean TDI), and LA indices, including dimensions, volume, ejection fraction, and LA strain components (reservoir, conduit, and contractile strain). Pulmonary artery systolic pressure (PASP) was also assessed as a measure of pulmonary hemodynamics. Additionally, right atrial (RA) size and right ventricular (RV) dimensions, volume, and function were recorded to provide further insight into atrial−ventricular interactions. All measurements were conducted in accordance with the latest guidelines of the European Association of Cardiovascular Imaging (EACVI) [20].
4. 24-h ECG ambulatory Holter monitoring: Study participants wore a 24-h ECG ambulatory Holter monitor to record continuous ECG data. Key measures included the total burden of premature atrial and ventricular contractions, episodes of AF, microvolt T-wave alternans (TWA), heart-rate variability (HRV) indices, and deceleration capacity.
5. Plasma biomarkers: Blood samples were collected at baseline and stored at −80 °C for subsequent biochemical analysis. The plasma biomarkers to be measured include galectin-3, homocysteine, high-sensitivity cardiac troponin I (hs-cTnI), procalcitonin, and brain natriuretic peptide (BNP).

#### 2.3.2. Follow-Up

Study participants will be followed up for 12 months after enrollment in the study. During this period, detailed records will be maintained for each patient, including the occurrence of new AF episodes and other MACE such as myocardial infarction, stroke, HF, and AF-associated hospitalizations. Any changes in medication will also be documented.

In the follow-up phase, participants will undergo a comprehensive re-evaluation, including a full standard transthoracic echocardiogram and 24-h ECG ambulatory Holter monitoring. The echocardiogram will assess the dimensions and functionality of the LV and LA, along with other parameters such as LVEF, GLS, diastolic indices, and RV function. The 24-h ECG ambulatory Holter monitoring will record continuous ECG data to measure parameters including the total burden of premature atrial and ventricular contractions, episodes of AF, microvolt TWA, HRV indices, and deceleration capacity.

The aim of this follow-up is to assess the feasibility of correlating changes in these echocardiographic and ECG parameters with the recurrence of AF. This will serve as an initial step toward evaluating whether such parameters could potentially predict arrhythmia relapses in a larger study cohort. By examining these associations, the study aims to explore potential factors contributing to AF recurrence and other cardiovascular outcomes, ultimately supporting the development of more personalized treatment strategies for patients with PAF.

### 2.4. Outcome Measures

The primary outcome measure is the recurrence of AF within 12 months from enrollment. This will be determined based on available electronic data and through detailed patient interviews during follow-up sessions. Secondary outcome measures will include a variety of clinical and diagnostic indicators, which will be used to evaluate the overall cardiovascular health of and AF progression in participants. These measures will encompass AF-related hospitalizations, types of cardioversions performed, the total burden of premature atrial and ventricular contractions, and episodes of AF recorded during 24-h ECG ambulatory Holter monitoring. Other ECG parameters such as microvolt TWA and HRV indices will also be assessed.

Additional secondary measures will include key performance metrics from CPET such as peak VO_2_ and VE/VCO_2_ slope, as well as echocardiographic indices like LA strain and LV GLS. Plasma biomarkers such as galectin-3, homocysteine, BNP, and hs-cTnI will also be measured to explore their potential role in predicting AF progression. The time to the first recurrence of AF will also be tracked, which may provide valuable insights into factors that could influence the recurrence of AF.

This study aims to assess the feasibility of monitoring and correlating these measures as potential predictors of AF recurrence, ultimately setting the stage for further validation in a larger, more definitive study.

### 2.5. Statistical Analysis

Data will be analyzed using appropriate statistical methods to evaluate the prognostic significance of CPET data, echocardiographic parameters, and plasma biomarkers. Descriptive statistics will be used to summarize quantitative and qualitative variables and to provide insights into baseline characteristics. Univariable and multivariable analyses will be performed to assess the association between these variables and the recurrence of AF, as well as related adverse outcomes. Various statistical models will be considered, including the following:

- Linear regression for the number of AF events during follow-up.
- Binary logistic regression for the occurrence of AF relapse during follow-up.
- Cox proportional hazards model for the time to an AF event during follow-up.

Given that the study population consists of generally healthy individuals without major comorbidities, the presence of significant confounders is expected to be minimal. However, to ensure robustness, multivariable models will include adjustments for relevant baseline clinical and demographic characteristics if such is deemed statistically or clinically important.

Sensitivity analyses will be conducted to assess the impact of missing data and potential biases. In particular, the missing data will be replaced, when appropriate, using targeted imputation techniques. The results using complete case analyses versus analyses based on multiple imputation will be compared, and the changes that are caused by the missing data will be evaluated. Subgroup analyses will be performed, stratified by key clinical variables (e.g., AF duration, CPET performance, and echocardiographic indices) to explore the consistency of findings across different patient profiles and possibly detect related bias. To further address bias, different modeling strategies will be considered to evaluate AF events during follow-up as already mentioned, including linear regression (targeting the number of AF events), binary logistic regression (targeting the occurrence of AF–Yes/No), and Cox regression (targeting the time to AF event). Nonparametric methods such as decision trees may be also employed. By using alternative model choices to assess AF events during follow-up, a broader understanding of the impacting factors will be achieved. Multicollinearity will be assessed and addressed in all multivariable analyses. Additionally, bootstrap resampling will be used by repeatedly performing model fitting on random subsets of data to assess stability.

For all models, the necessary assumptions will be evaluated and appropriate adjustments will be made. A combination of these analytical approaches will enhance the understanding of the data and improve the robustness of the study findings.

## 3. Discussion

The PLACEBO pilot study aims to explore novel predictors of AF recurrence, with the primary outcome measure being relapse of AF within 12 months from study enrollment. Secondary outcome measures include a range of clinical and diagnostic indicators such as AF-related hospitalizations, cardioversions, the total burden of premature atrial and ventricular contractions, and various ECG parameters. Our findings aim to highlight the complexity of AF management and the potential benefits of a multifaceted approach to predicting AF recurrence.

Cardiorespiratory fitness has been a contentious topic in AF research. Numerous studies have suggested that higher levels of fitness correlate with a reduced incidence of AF and improved prognosis [21]. These studies indicate that increased physical activity and better cardiorespiratory health can lead to favorable cardiovascular adaptations, potentially reducing the burden of AF. However, contrasting studies have revealed an intriguing paradox: individuals with high fitness levels, particularly endurance athletes, exhibit a higher prevalence of AF [22]. This increased risk is thought to be related to physiological changes associated with intensive training, such as atrial enlargement, increased inflammation and thus fibrosis, and heightened vagal tone, which may all predispose to AF [23]. The PLACEBO pilot study contributes to this ongoing field by examining the relationship between fitness levels and AF recurrence in a diverse patient population, aiming to provide a more nuanced perspective on how fitness impacts AF prognosis.

In our cohort, the integration of CPET parameters, echocardiographic indices, and plasma biomarkers will provide a comprehensive dataset to analyze. CPET metrics such as peak VO_2_ and the VE/VCO_2_ slope may offer valuable insights into the cardiorespiratory efficiency of patients. These measures are key for understanding physical capacity and cardiovascular health in individuals with AF. Echocardiographic measurements, including LA strain and LV GLS, will enrich our analysis by offering detailed information on cardiac structure and function. Atrial remodeling plays a crucial role in the pathophysiology of AF, with structural and functional changes influencing recurrence risk. The aforementioned echocardiographic indices provide insights into atrial function and fibrosis, both of which contribute to AF persistence. Prior research has shown that extensive atrial ablation can lead to structural remodeling and LA stiff syndrome, which may affect diastolic function and clinical outcomes [24]. While our study excludes patients with prior AF ablation to maintain a homogeneous population, these findings highlight the broader implications of atrial remodeling for AF prognosis.

Emerging evidence suggests that AF pathophysiology is influenced not only by LA substrate but also by RA and RV function, particularly in older adults or those with pulmonary hypertension [25]. Studies indicate that elevated right heart pressures and RA dysfunction may contribute to AF recurrence in select populations, particularly those with COPD, pulmonary arterial hypertension, or RV overload [26]. Besides LA function, we have incorporated in our study right heart echocardiographic markers, including RA size, RV function, and pulmonary pressures, to explore potential associations with AF recurrence. While our sample size is not designed for formal age-based subgroup analyses, these parameters may provide preliminary insights into whether right heart dysfunction contributes to AF recurrence even in a generally healthy cohort.

Additionally, recent studies suggest that AF recurrence and outcomes may differ across distinct clinical phenotypes [27]. Given that our study includes a broad age range and does not impose strict exclusions based on comorbidities, it may provide useful data on AF recurrence patterns across different phenotypic subgroups. These findings could help in generating hypotheses for future research aimed at refining predictive models for AF recurrence based on both left and right heart contributions to arrhythmogenesis. Notably, the prevalence of heart failure with preserved ejection fraction (HFpEF) is rising and is expected to increase further in the coming years; AF is highly prevalent in HFpEF populations, with registry data indicating its presence in at least 50% of patients [28]. Since HFpEF is frequently associated with RV dysfunction and structural remodeling, our study may provide important prognostic insights for this specific population [29].

Finally, plasma biomarkers like galectin-3, homocysteine, hs-cTnI, and BNP could play a role in assessing underlying inflammatory and cardiac stress processes, which are critical to the pathophysiology of AF [30,31,32,33,34]. Galectin-3, in particular, has been implicated in inflammatory and fibrotic pathways that contribute to atrial remodeling and AF development; elevated galectin-3 levels have been associated with atrial fibrosis, increased LA stiffness, and worse clinical outcomes in patients with AF, highlighting its potential as a biomarker for disease progression and a possible therapeutic target [35,36].

Given the pilot nature of this study, the results will provide preliminary insights into the potential predictors of AF recurrence. The findings could help clinicians better stratify patients based on risk, enabling more tailored and effective interventions. This personalized approach may lead to improved outcomes by preventing AF episodes and reducing associated morbidity. Moreover, the study highlights the importance of comprehensive follow-up in AF patients, which could facilitate early detection of recurrence and prompt management. Future, larger-scale studies are needed to validate these preliminary findings and incorporate them into standard clinical practice.

### Study Limitations

This pilot study has a series of limitations that should be considered when interpreting the results. One key limitation is the small sample size, which, typical for pilot studies, limits the statistical power and generalizability of the findings. Consequently, the results should be viewed as exploratory and cannot be conclusively applied to the broader population of patients with PAF.

Additionally, the study lacks an independent validation cohort, which limits the ability to externally validate any potential predictive models derived from this dataset. However, as a feasibility study, the primary objective is to identify potential signals and generate hypotheses, rather than to develop definitive prediction models at this stage. Future large-scale studies will be necessary to validate these findings in independent cohorts and refine predictive models before clinical implementation.

Another limitation is the relatively short 12-month follow-up period, which may not be sufficient to capture the long-term dynamics of AF recurrence and other cardiovascular outcomes, with longer-term studies being necessary to better understand the chronicity of these events.

Despite these limitations, the PLACEBO pilot study serves as an important first step toward evaluating the feasibility of a multifaceted approach to predicting AF recurrence, setting the scene for future, larger-scale studies to confirm and refine these findings.

## 4. Conclusions

The PLACEBO pilot study seeks to provide valuable insights into the potential predictors of AF recurrence, focusing on the importance of a comprehensive, multifaceted approach that integrates CPET data, echocardiographic indices, and plasma biomarkers. Although preliminary, this study lays the groundwork for more extensive investigations into how these parameters can be used to predict AF recurrence and guide personalized management strategies. The findings, while exploratory, suggest that such a comprehensive approach could significantly improve risk stratification and clinical decision-making for patients with PAF. Further research with larger, more diverse populations and extended follow-up is needed to confirm these findings and refine the methods for practical application in clinical settings. This pilot study represents an important first step toward enhancing our understanding of AF and optimizing strategies for patient care.

## Data Availability

Data are contained within the article.
